# Is physical exercise associated with reduced adolescent social anxiety mediated by psychological resilience?: evidence from a longitudinal multi-wave study in China

**DOI:** 10.1186/s13034-025-00867-8

**Published:** 2025-03-05

**Authors:** Jingtao Wu, Yanhong Shao, Wanli Zang, Jun Hu

**Affiliations:** 1 School of Physical Education, Leshan Normal University, 778 Binhe Road, Shizhong District, Leshan, 614000 Sichuan China; 2 Xiangshui Teacher Development Center, Yancheng, China; 3 Postgraduate School, Harbin Sport University, Harbin, China

**Keywords:** Physical exercise, Adolescents, Psychological resilience, Social anxiety, Longitudinal, Cross-Lagged, Association

## Abstract

**Objective:**

The study aims to investigate whether physical exercise is associated with psychological resilience, thereby significantly affecting adolescent social anxiety, and to analyze the longitudinal cross-temporal stability between these three interrelated factors.

**Methods:**

The methodology involved a survey utilizing the International Physical Activity Questionnaire (IPAQ), the Connor-Davidson Resilience Scale (CD-RISC), and the Social Anxiety Scale (SAS) across various regions in China, including Sichuan, Guangdong, Shanxi Province, and Beijing. A total of 1259 participants were recruited from primary, middle, and high schools, with an average age of 13.7 years. The sample comprised 626 males and 633 females. A longitudinal tracking survey approach was implemented, commencing in June 2023, with follow-up rounds scheduled every three months, culminating in a total of four rounds.

**Results:**

The results are as follows: (1) Physical exercise was significantly positively correlated with psychological resilience (r = 0.35, p < 0.001) and significantly negatively correlated with social anxiety (r = − 0.26, p < 0.001); (2) Physical exercise could significantly negatively predict social anxiety in the next period (PET1 → SAT2: β = − 0.31, p < 0.001); (3) Psychological resilience played a mediating role in the association between physical exercise on social anxiety (PET1 → PRT2: β = 0.42, PRT2 → SAT3: β = − 0.38, p-values < 0.001).

**Conclusion:**

Physical exercise, psychological resilience, and social anxiety exhibit cross-temporal stability, and physical exercise has a significant lagged effect on psychological resilience and social anxiety. Physical exercise may indirectly reduce social anxiety through its association with enhanced psychological resilience in adolescents.

## Introduction

In recent years, social anxiety among adolescents has drawn significant attention and research interest [1, 2]. Defined as a common psychological issue involving concerns [3], discomfort [4], and fear in public settings [5], social anxiety is a prevalent and often hidden disorder [6], especially during adolescence—a critical period for socialization [7] and identity development [8]. Sensitive to social approval [9] and interactions [10], adolescents with social anxiety face mental health challenges [11], academic difficulties [12], strained peer relationships [13], and reduced social support [14]. Therefore, alleviating social anxiety in adolescents holds great historical and practical importance.

Researchers are increasingly exploring factors influencing adolescent social anxiety, particularly the role of physical exercise as an intervention [15–17]. Studies indicate that physical exercise can reduce negative emotions such as anxiety [18, 19], depression [20, 21], and stress [22, 23] in adolescents. It also boosts self-confidence [24, 25] and self-efficacy [26, 27], indirectly improving emotional regulation [28, 29]. The positive impact of physical activity is notably significant in adolescents.

On one hand, physical exercise significantly bolsters physiological mechanisms, rapidly enhances cardiopulmonary function, and modulates neuroexpression mechanisms, including increasing hormone levels such as endorphins and dopamine, which are instrumental in effectively reducing anxiety [30]. Scholars have confirmed that regular engagement in physical activities can effectively lower cortisol levels [31], thereby biologically suppressing the expression of emotions like anxiety and depression [32]. On the other hand, physical exercise facilitates more opportunities for communication and interaction, fostering the establishment of positive interpersonal relationships [33]. This leads to greater social approval and recognition from peers, which is conducive to emotional resonance and communication, and thus alleviates internal tension [34]. Through regular participation in sports activities, adolescents can gain social approval and recognition, diminishing the impact of negative social evaluations.

In addition to the direct impact of physical exercise on adolescent social anxiety, psychological resilience, as a key psychological factor, also plays an important mediating role in this process. Psychological resilience is not only the ability of individuals to cope with adversity, stress, or setbacks but also an important factor affecting social anxiety [12]. Studies have shown that psychological resilience can help individuals alleviate anxiety in social situations, enhance self-efficacy, and improve emotional regulation capabilities [35]. Especially in adolescents, psychological resilience can effectively alleviate psychological pressure from the social environment, allowing them to maintain emotional stability in the face of negative evaluations and reduce social avoidance behaviors [36]. More importantly, psychological resilience is considered a significant mediating mechanism in the impact of physical exercise on social anxiety. Existing research has pointed out that adolescents who regularly participate in physical exercise not only benefit physiologically but also enhance psychological resilience through exercise, thereby better coping with social pressure [37]. This process is achieved by improving adolescents’ self-efficacy and emotional regulation capabilities [38], thereby reducing the occurrence of social anxiety and avoidance behaviors [39].

While numerous studies have documented the positive impact of physical exercise on positive emotions, most are cross-sectional, neglecting the need for longitudinal research to understand the dynamic interplay between exercise, psychological well-being, and social anxiety in adolescents. Addressing this gap, our study builds on prior research to elucidate how physical exercise influences adolescent social anxiety, with a particular focus on the mediating effects of psychological resilience, self-efficacy, and emotional regulation, aiming to enhance theoretical and practical insights into interventions for adolescent social anxiety.

### The relationship between physical exercise and social anxiety

Research has shown that adolescents who engage in physical exercise tend to exhibit lower levels of anxiety in social contexts, providing theoretical support for this study [40, 41]. Physical exercise impacts individual social anxiety through various mechanisms. Firstly, physical exercise alleviates anxiety through physiological mechanisms. Studies have indicated that physical exercise can regulate the secretion of neurotransmitters like endorphins and dopamine, which enhance mood and decrease anxiety [18]. Additionally, exercise is known to reduce cortisol levels, the hormone linked to stress and anxiety, thereby equipping adolescents to manage social pressures more effectively [42].

Secondly, physical exercise provides adolescents with more social opportunities, as studies indicate that adolescents participating in team sports can establish positive peer relationships through cooperation and competition, significantly aiding in alleviating their discomfort in social situations [34, 43]. Within team sports, adolescents not only build these relationships but also enhance their self-confidence in social interactions, contributing to a reduction in social anxiety. Additionally, physical exercise provides a secure social setting that encourages adolescents to engage in interactions without the intense pressure of social judgment [44]. This experience can help adolescents gradually overcome social anxiety and develop confident social behaviors.

Furthermore, physical exercise is known to bolster self-efficacy in individuals. Studies reveal that adolescents who participate in physical activities display greater self-efficacy and emotional regulation when confronting social stress, enabling them to handle social challenges with more effectiveness [45, 46]. The sense of achievement and progress from physical exercise further enhances adolescents’ self-efficacy, which in turn empowers them to engage in social situations with increased confidence, reducing the likelihood of social anxiety. Indeed, research indicates that adolescents who are physically active possess higher self-efficacy and emotional regulation skills, which equip them to confront social stress more resiliently and diminish their anxious reactions [39].

Although existing studies have shown that physical exercise reduces adolescent social anxiety, most research still focuses on cross-sectional designs that assess the relationship at a single point, neglecting the long-term effects [47]. For example, scholars have found through cross-sectional studies that adolescents who participate in physical exercise show lower levels of social anxiety than their peers who do not exercise [48]. However, the study cannot reveal the long-term causal relationship between physical exercise and social anxiety, especially how physical exercise affects the changing trends of adolescent social anxiety. Therefore, this study uses physical exercise as the independent variable and leveraging longitudinal data to explore the development and mechanisms of impact on adolescent social anxiety. This approach will help reveal the causal relationships between physical activity and social anxiety, filling a gap in existing literature [49]

### The mediating role of psychological resilience

Self-Determination Theory [43] posits that meeting individuals’ needs for autonomy, competence, and relatedness is pivotal for psychological development, directly influencing psychological functioning and behavioral performance [50]. Evidence suggests that satisfying these needs through physical activity can boost psychological resilience, a key factor in managing stress and anxiety among adolescents [51]. This is particularly pertinent as studies show that resilient adolescents are more adept at handling social pressures [52]. As a constructive external activity, physical exercise can enhance psychological resilience and mitigate social anxiety by fulfilling these core psychological needs. Regular engagement in physical activity not only meets these needs but also strengthens self-efficacy, a crucial component of psychological resilience [53].

Psychological resilience is the capacity to maintain mental balance and swiftly recover from stress and challenges, making it an essential psychological resource for managing social anxiety [54]. Studies consistently demonstrate that individuals with greater psychological resilience report lower levels of social anxiety, which supports our theoretical framework [55]. This resilience is frequently cultivated through adversarial experiences, such as surmounting obstacles in sports [56]. Consequently, physical exercise could significantly mitigate social anxiety by bolstering adolescents' psychological resilience. Consistent research indicates that those with higher resilience face less social anxiety, implying that resilience-enhancing interventions through physical activity are advantageous [57]. Such resilience is often developed through experiences that strengthen one’s capacity to confront adversity, including overcoming challenges in sports and other physical activities [58].

Self-Determination Theory posits that physical activity is instrumental in fulfilling the basic psychological needs for autonomy, competence, and relatedness, which are essential for bolstering psychological resilience and managing stress and anxiety in adolescents [59]. This link is underscored by research showing that self-efficacy acts as a mediator between physical activity and resilience [60]. Adolescents engaging in physical exercise experience a sense of autonomy and control by choosing sports that suit them, thereby strengthening their self-efficacy [40], which in turn enhances their ability to navigate social scenarios—a key aspect of resilience [61]. This enhanced self-efficacy makes them more confident in social situations and reduces anxiety stemming from the uncertainty of social contexts. Studies also show that increased self-efficacy correlates with lower levels of anxiety in social interactions, reinforcing the importance of physical activity as a strategy for anxiety reduction [62]. Moreover, physical challenges in exercise help adolescents build competence, a significant factor in diminishing social anxiety [63]. This competence contributes to resilience and underscores the importance of physical activity for psychological well-being [19].

Physical exercise also furnishes adolescents with ample opportunities for social connections, facilitating the development of positive relationships and support networks. These social connections are vital for building resilience, as they offer emotional support and a sense of belonging, which are crucial in mitigating social anxiety [64]. These social connections are also vital for building resilience, as they offer emotional support and a sense of belonging, which are crucial in mitigating social anxiety [65]. The satisfaction of relatedness needs helps individuals gain emotional support and a sense of belonging through interactions with peers, thereby enhancing psychological resilience. This relational aspect of resilience is supported by research indicating that social support is a key factor in managing anxiety [66]. In team sports, adolescents enhance their resilience to social pressure and anxiety through cooperative and competitive peer interactions [67]. This interaction bolsters resilience as individuals master social challenges [55]. Consequently, physical activity not only boosts adolescents’ psychological resilience by increasing self-efficacy and competence but also mitigates social anxiety by promoting social bonding.

The Compensatory Theory posits that unmet fundamental psychological needs drive individuals to seek alternative means of compensation [68]. Physical exercise serves as a constructive outlet for adolescents, fulfilling their needs for autonomy, competence, and relatedness, which in turn bolsters their psychological resilience and diminishes social anxiety [69]. Essentially, engaging in physical activity mitigates social anxiety by addressing these core psychological requirements.

Researchers exploring psychological resilience as a mediator have noted its dynamic mechanisms, often overlooked in past studies that focused on resilience levels at a single point rather than its developmental progression over time [70]. The starting level and rate of change in resilience could differentially influence the link between physical exercise and social anxiety. Adolescents engaging in physical exercise may begin with higher resilience, aiding in managing social stress and anxiety [71]. Continued exercise might accelerate resilience, thereby amplifying its anxiety-reducing impact, indicating a dynamic interplay needing further study [72]. Thus, to fully understand the mediation of resilience between physical exercise and social anxiety, both initial resilience levels and the velocity of its development must be assessed.

### Research hypotheses

Based on the aforementioned theories and research, this study proposes the following three hypotheses:

#### H1

Physical exercise (PE), adolescent psychological resilience (PR), and social anxiety (SA) exhibit longitudinal cross-temporal stability at different time points.

#### H2

Physical exercise (PE) can significantly predict adolescents’ social anxiety (SA) in the next period.

#### H3

Psychological resilience (PR) mediates the relationship between physical exercise (PE) and adolescent social anxiety (SA).

**Fig. 1:**
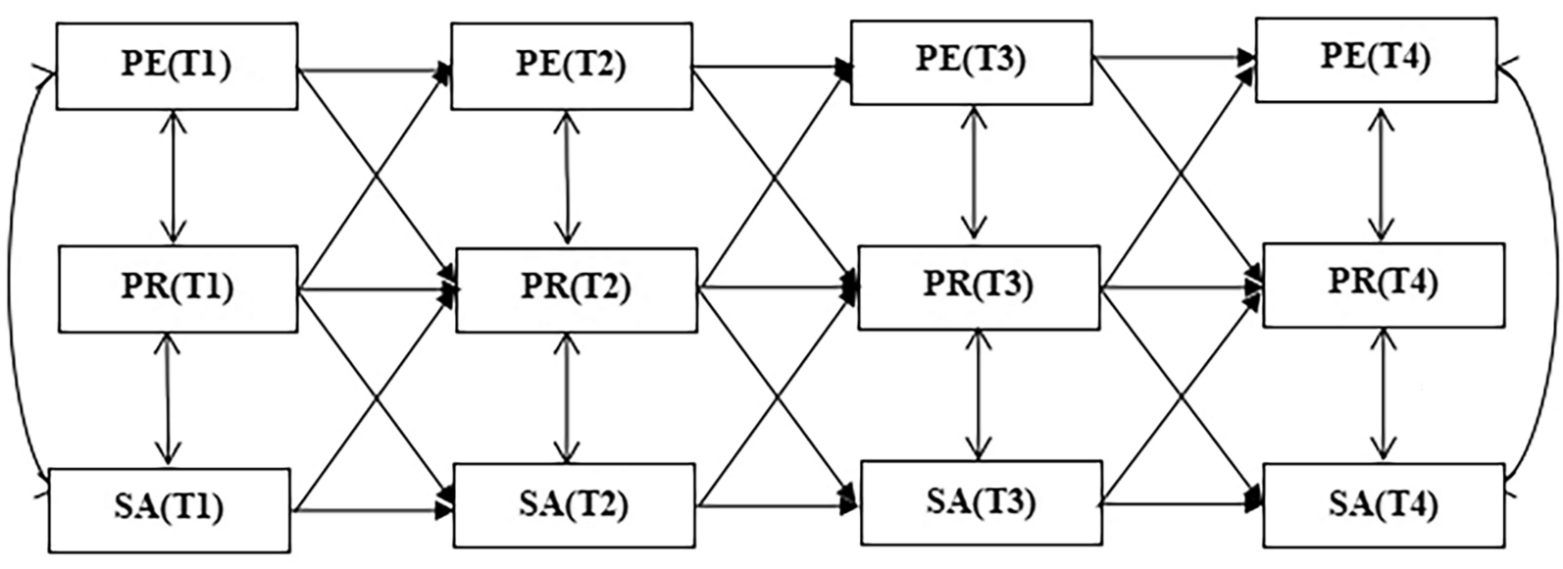
Physical Exercise abbreviated as PE, Social Anxiety abbreviated as SA, Psychological Resilience abbreviated as PR

To visually represent these hypotheses, a theoretical model can be constructed as depicted in Fig. 1.

## Research methodology

### Participants

In this study, a stratified sampling approach was utilized to account for the cultural, economic, and regional diversities across China, focusing on central, western, eastern, and northern regions, including cities such as Beijing, Guangzhou, Leshan, Xi'an, and Zhanjiang. The aim was to survey primary, middle, and high school students from these areas. For each survey round, two classes were randomly selected from each grade in each school, and 30 students were chosen at random from each class to ensure a diverse and representative sample. Prior to questionnaire distribution, contact was established with local coordinators, psychological counselors, questionnaire explainers, homeroom teachers, and principals to facilitate the research process. A pilot test was conducted to ensure the questionnaires’ validity, and revisions were made based on feedback from the student participants.

Informed consent was obtained from all participants’ parents or guardians before the study began. An informational session was held to explain the study’s objectives, procedures, and potential risks, emphasizing the importance of their involvement. Parents or guardians provided their consent before each data collection phase. The study was granted ethical approval by the Institutional Review Board prior to initiation.

To mitigate homogeneity bias from repeated measurements, the sequence of scales within the questionnaires was altered for each survey. Homeroom teachers and school-appointed survey coordinators determined which student IDs to exclude due to psychological distress or physical disabilities, ensuring the consistency of each participant’s ID across the four follow-up surveys.

During each questionnaire distribution, homeroom teachers, questionnaire interpreters, and psychological counselors were on hand to supervise and assist students in completing the questionnaires. In the initial survey conducted in September 2023, 1500 questionnaires were distributed, and 1487 valid responses were received. Subsequent surveys were carried out every three months. By the conclusion of the fourth survey round, 228 students had withdrawn from the study due to reasons such as transferring schools or suspension, resulting in a 15.2% dropout rate. The final valid sample comprised 1259 participants, including 403 primary school students (32%), 457 middle school students (36.3%), and 399 high school students (31.7%); 626 males (49.7%) and 633 females (50.3%), with an average age of 13.7 ± 2.5 years. The sample attrition did not exhibit structural bias, and there were no significant differences in physical activity frequency, psychological resilience, and social anxiety across the four surveys (t (1259) = − 0.45, p = 0.65), indicating the sample's representativeness and stability.

### Measures and procedures

In this study, physical exercise (PE), psychological resilience (PR), and social anxiety (SA) were measured using validated scales.

#### Physical activity questionnaire

The International Physical Activity Questionnaire (IPAQ), tailored for adolescents, was used to measure participants’ exercise levels. The IPAQ includes 7 items on the frequency and duration of moderate and vigorous physical activities, as well as walking. The scale has been validated internationally and is recognized for its reliability in measuring physical activity across diverse populations [73]. The IPAQ includes seven items that assess the frequency and duration of walking, moderate-intensity, and vigorous-intensity activities. The scoring method sums up all physical activities over the past seven days, specifically calculated as (walking days × walking minutes × 3.3) + (moderate-intensity days × moderate-intensity minutes × 4) + (vigorous-intensity days × vigorous-intensity minutes × 8).The total score for the IPAQ ranges from 0 to 28, with higher scores indicating greater levels of physical activity. The scale in the study showed a good fit with the data (χ^2^/df = 3.78, GFI = 0.91, AGFI = 0.90, RMSEA = 0.058), and the Cronbach’s α coefficients at four time points were 0.88, 0.86, 0.84, and 0.82, respectively.

#### Psychological resilience scale

The Connor-Davidson Resilience Scale (CD-RISC) was used to measure individual levels of psychological resilience (PR). Originally developed by Australian scholars [74] and subsequently adapted into a Chinese version by Chinese researchers, the CD-RISC has become a staple in adolescent resilience studies [70]. The scale consists of 25 items across 5 dimensions: positive coping, goal orientation, control, tolerance of stress, and social support. Responses are measured on a 5-point Likert scale, from 1 “never” to 5 “always”. Scores on the CD-RISC range from 25 to 125, with higher scores indicating higher levels of psychological resilience. The scale was slightly modified to align with the cognitive abilities and life experiences of adolescents. It exhibited a good fit with the data (χ^2^/df = 4.22, GFI = 0.92, AGFI = 0.91, RMSEA = 0.061), attesting to its structural validity and reliability. The Cronbach’s α coefficients for the CD-RISC across four time points were 0.90, 0.89, 0.87, and 0.85, respectively.

#### Social anxiety scale for adolescents

The Social Anxiety Scale for Adolescents (SAS-A) was used to measure the level of social anxiety among adolescents. Originally developed by Finnish scholars [75] and later tested for invariance in the United States, Spain, and China by Torregrosa and colleagues, showing good psychometric properties [76]. The scale consists of 18 items across 3 dimensions: Fear of Negative Evaluation (FNE), Social Avoidance and Distress (SAD), and Anxiety in New Social Situations (ANS). It uses a 5-point Likert scale, ranging from 1 “not at all” to 5 “very much,” with higher scores indicating higher levels of social anxiety. The total score for the SAS-A ranges from 18 to 90, with higher scores indicating higher levels of social anxiety. Certain items were culturally adapted to ensure suitability for Chinese adolescents. The three-factor model in the study showed a good fit with the data (χ^2^/df = 3.65, GFI = 0.93, AGFI = 0.92, RMSEA = 0.057), indicating good structural validity and reliability. The Cronbach’s α coefficients for the SAS-A at four different time points were 0.89, 0.91, 0.88, and 0.86, respectively.

### Ethical approval

All procedures related to this study strictly adhered to the ethical principles of the Declaration of Helsinki and complied with legal regulations and the institutional guidelines of Leshan Normal University. The study has been granted ethical approval by the Academic Committee of Leshan Normal University (Approval No.: LSNU20230615), with ethical oversight provided by the committee. Written informed consent was obtained from both guardians and participants before the study, and strict protection of participants’ privacy and personal information was ensured throughout the research process, meeting ethical requirements and confidentiality standards.

### Data analysis

Data were initially entered into SPSS 26.0 to calculate correlation coefficient matrices across four time points, assessing the relationships between physical exercise, psychological resilience, and social anxiety. This was followed by structural equation modeling in Mplus 8.0, which was executed in five steps: (1) Correlation coefficient matrix analysis was conducted to gauge the static correlations among the three variables at various time points, establishing preliminary variable interrelationships; (2) A cross-lagged panel model (CLPM) was established for physical exercise and adolescent social anxiety to determine the predictive power of physical exercise on social anxiety at different time points and to affirm its longitudinal influence; (3) A CLPM was formulated for physical exercise and psychological resilience to investigate the predictive significance of physical exercise on changes in psychological resilience over time and to elucidate its lagged effects.

(4) A three-variable CLPM was developed for physical exercise, psychological resilience, and social anxiety to dissect their cross-lagged relationships and to explore the mediating role of psychological resilience in the impact of physical exercise on social anxiety;

(5) Mediation effect testing was performed using the Bootstrap method to assess the mediating role of psychological resilience between physical exercise and social anxiety [64]. A non-zero 95% confidence interval indicates a significant mediating effect. All models were estimated using full information maximum likelihood [77], with model fit indices including χ^2^/df, GFI, AGFI, CFI, TLI, and RMSEA to ensure a good fit between the model and the data.

### Research process

**Fig. 2:**
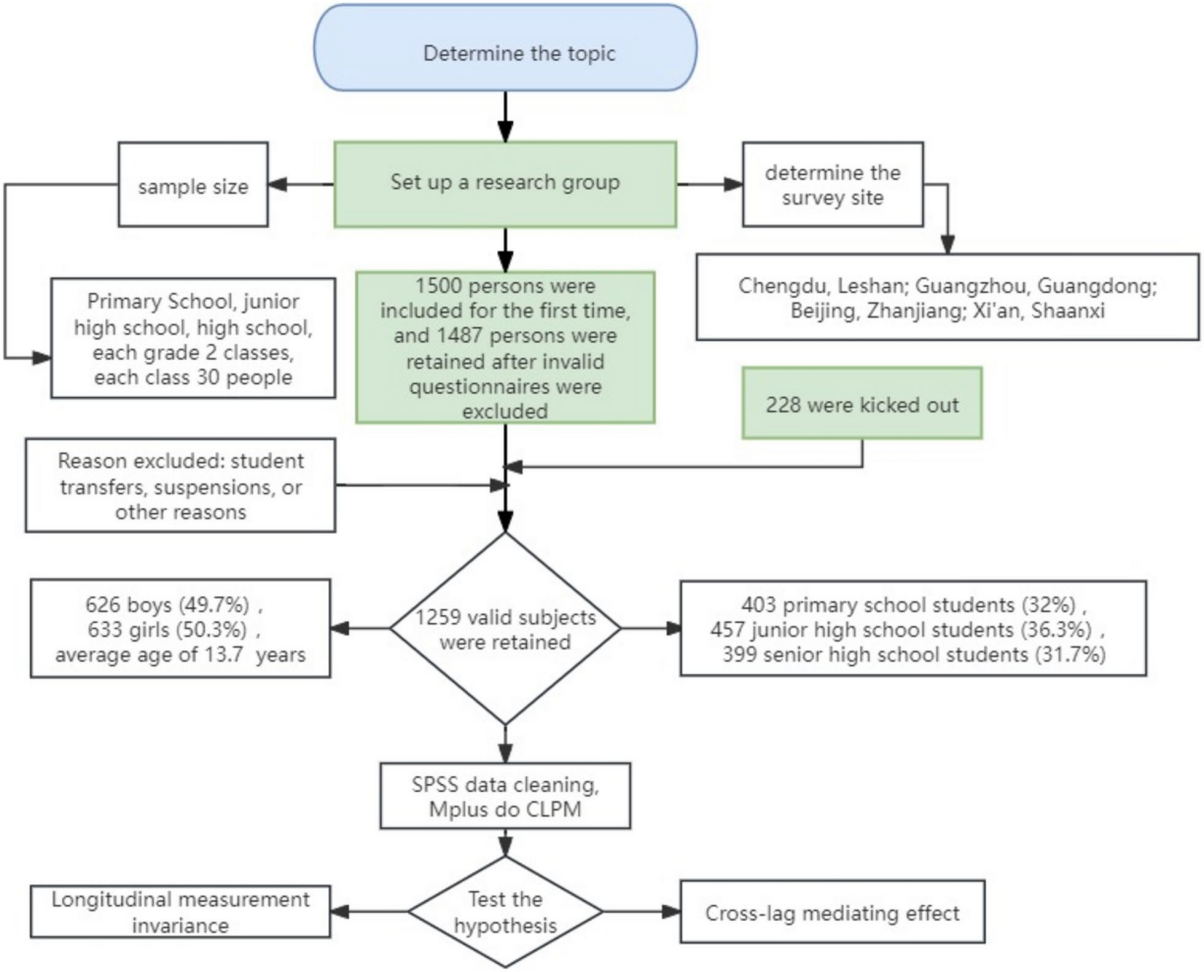
Research flowchart

This study employed a meticulous scientific methodology, segmented into distinct phases: Initially, cluster sampling selected participants from primary and secondary school students across various regions, followed by a preliminary screening. Subsequently, four follow-up assessments were implemented at set intervals, with questionnaire order randomized to mitigate homogeneity bias. Data collection was succeeded by entry and preprocessing in SPSS, and the construction of cross-lagged panel models (CLPM) in Mplus 8.0 to analyze the interplay between physical exercise, psychological resilience, and social anxiety, as well as to evaluate the mediating role of psychological resilience. Throughout the analysis, stringent model fit criteria were upheld to ensure model-data congruence. Ethical protocols were strictly observed, with informed consent and privacy safeguards in place for participants, thereby ensuring the study’s legality and scientific integrity. A flowchart of the study is depicted in Fig. 2.

## Results and analysis

### Test for homogeneity of variances of observed variables

Table 1 summarizes the effects of origin, age, and gender on physical exercise (PE), psychological resilience (PR), and social anxiety (SA). Urban students scored higher in PE (22.53 ± 0.89) and PR (78.77 ± 0.79) compared to rural students (PE: 20.43 ± 0.97, PR: 76.75 ± 0.78). However, rural students had lower SA (32.03 ± 0.67) than urban students (34.15 ± 0.65). Among the 10-year-olds, PE averaged 21.50 ± 0.50, PR was 80.62 ± 0.33, and SA was 29.05 ± 0.62, indicating higher resilience and lower anxiety. As age increased, PE decreased, with the highest SA (34.12 ± 0.64) at age 12. Males outperformed females in PE (22.67 ± 0.92 vs. 20.28 ± 0.92) but had lower SA (32.03 ± 0.65 vs. 35.13 ± 0.66). No significant gender difference was found in PR (males: 78.77 ± 0.23, females: 76.02 ± 0.78). These findings highlight how origin, age, and gender affect mental health and physical activity.

**Table 1:** Variance test

Variable	Category	PE	PR	SA
Place of origin	City	22.53 ± 0.89	78.77 ± 0.79	34.15 ± 0.65
	Countryside	20.43 ± 0.97	76.75 ± 0.78	32.03 ± 0.67
	*T*	4.574	9.333	7.086
	*p*	0.010^*^	0.000^**^	0.001^**^
Age	*10* years old	21.50 ± 0.50	80.62 ± 0.33	29.05 ± 0.62
	11 years old	24.13 ± 0.11	80.35 ± 0.18	30.22 ± 0.10
	12 years old	22.57 ± 0.80	76.66 ± 0.68	32.09 ± 0.23
	13 years old	21.48 ± 0.98	79.79 ± 0.81	34.12 ± 0.64
	14 years old	19.25 ± 1.02	81.81 ± 0.92	30.89 ± 0.66
	15 years old	17.75 ± 1.33	74.50 ± 0.51	28.75 ± 0.44
	*F*	7.282	4.071	6.1
	*p*	0.000^**^	0.003^**^	0.000^**^
Gender	Male	22.67 ± 0.92	78.77 ± 0.23	32.03 ± 0.65
	Female	20.28 ± 0.92	76.02 ± 0.78	35.13 ± 0.66
	*T*	75.221	1.618	8.183
	*p*	0.000^**^	0.204	0.004^**^

Physical Exercise is abbreviated as PE, Social Anxiety as SA, and Psychological Resilience as PR

* indicates *P* < 0.05, ** indicates *P* < 0.001

### Correlation coefficient matrix

Table 2 displays the correlation coefficients for Physical Exercise PE(T1)-PE(T4), Psychological Resilience PR(T1)-PR(T4), and Social Anxiety (SAT1-SAT4) at different time points. Physical Exercise shows significant cross-temporal stability, with correlation coefficients of 0.418, 0.359, and 0.362 between PE(T1) and PE(T2), PE(T3), PE(T4), respectively (p < 0.001).The correlations among the periods for Psychological Resilience are also high, with correlation coefficients of 0.421, 0.385, and 0.350 between PR(T1) and PR(T2), PR(T3), PR(T4), respectively (p < 0.001). Social Anxiety exhibits significant correlations across periods, with correlation coefficients of 0.269, 0.235, and 0.299 between SA(T1) and SA(T2), SA(T3), SA(T4), respectively (p < 0.001). Additionally, Physical Exercise is significantly positively correlated with Psychological Resilience, as indicated by the correlation coefficient of 0.351 between PE(T1) and PR(T1) (p < 0.001), suggesting that greater Physical Exercise is associated with higher Psychological Resilience; Physical Exercise is significantly negatively correlated with Social Anxiety, with a correlation coefficient of − 0.261 between PE(T1) and SA(T1) (p < 0.001), indicating that greater Physical Exercise is associated with lower Social Anxiety. Psychological Resilience is also negatively correlated with Social Anxiety, with a correlation coefficient of − 0.262 between PR(T1) and SA(T1) (p < 0.001), further supporting the mediating role of Psychological Resilience between Physical Exercise and Social Anxiety.

**Table 2:** Correlation coefficient matrix

	*M*	*SD*	1	2	3	4	5	6	7	8	9	10	11	12
PE(T1)	3.259	0.740	1											
PE(T2)	3.238	0.747	0.418**	1										
PE(T3)	3.167	0.752	0.359**	0.383**	1									
PE(T4)	3.250	0.775	0.362**	0.419**	0.452**	1								
PR(T1)	3.055	0.633	0.351**	0.408**	0.364**	0.343**	1							
PR(T2)	3.057	0.646	0.398**	0.353**	0.382**	0.403**	0.421**	1						
PR(T3)	3.021	0.667	0.353**	0.385**	0.361**	0.435**	0.385**	0.415**	1					
PR(T4)	3.047	0.667	0.350**	0.421**	0.447**	0.355**	0.358**	0.435**	0.465**	1				
SA(T1)	2.709	1.980	− 0.261**	− 0.131**	− 0.131**	− 0.148**	− 0.262**	− 0.127**	− 0.137**	− 0.139**	1			
S(AT2)	2.840	1.994	− 0.176**	− 0.242**	− 0.141**	− 0.160**	− 0.175**	− 0.257**	− 0.138**	− 0.178**	0.269**	1		
SA(T3)	2.801	1.991	− 0.139**	− 0.205**	− 0.260**	− 0.191**	− 0.151**	− 0.201**	− 0.248**	− 0.197**	0.235**	0.276**	1	
SA(T4)	2.674	1.974	− 0.190**	− 0.193**	− 0.185**	− 0.300**	− 0.191**	− 0.193**	− 0.182**	− 0.313**	0.299**	0.306**	0.358**	1

Physical Exercise is abbreviated as PE, Social Anxiety as SA, and Psychological Resilience as PR

* indicates *P* < 0.05, ** indicates *P* < 0.001

### Longitudinal cross-lagged effects between physical exercise and social anxiety

**Fig. 3:**
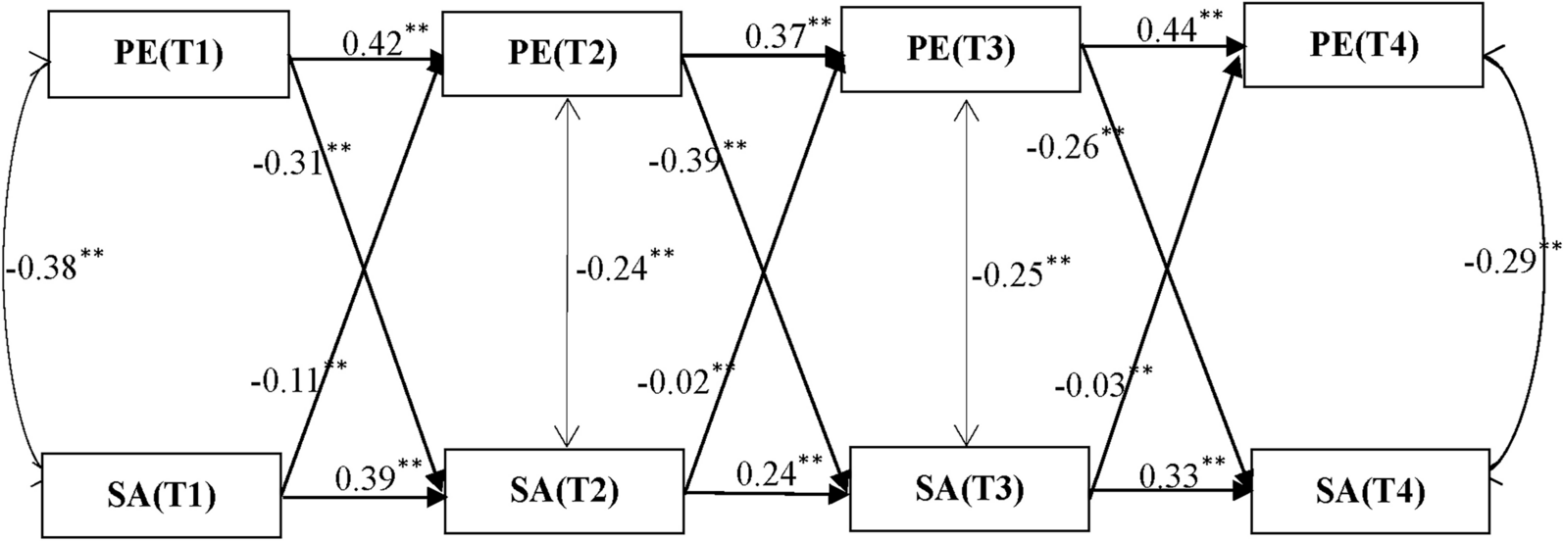
Cross-Lagged Effects between Physical Exercise and Social Anxiety. Physical Exercise abbreviated as PE, Social Anxiety abbreviated as SA, Psychological Resilience abbreviated as PR

Figure 3 displays the cross-lagged effects between physical exercise and social anxiety. Firstly, the negative predictive effect of physical exercise on social anxiety is significant in all periods, showing a stable influence. Specifically, the path coefficient from PE(T1) to SA(T2) is − 0.31, from PE(T2) to SA(T3) is − 0.39, and from PE(T3) to SAT4 is − 0.26, indicating that the more adolescents engage in physical exercise at earlier periods, the lower their subsequent levels of social anxiety, confirming the significant alleviating effect of physical exercise on social anxiety. In contrast, the reverse predictive effect of social anxiety on physical exercise is weaker. The path coefficient from SA(T1) to PE(T2) is − 0.11, from SA(T2) to PE(T3) is − 0.02, and from SA(T3) to PE(T4) is − 0.03, suggesting that although Social Anxiety has a certain inhibitory effect on physical exercise, the impact is minimal.

### Longitudinal cross-lagged effects among physical exercise, psychological resilience, and social anxiety

**Fig. 4:**
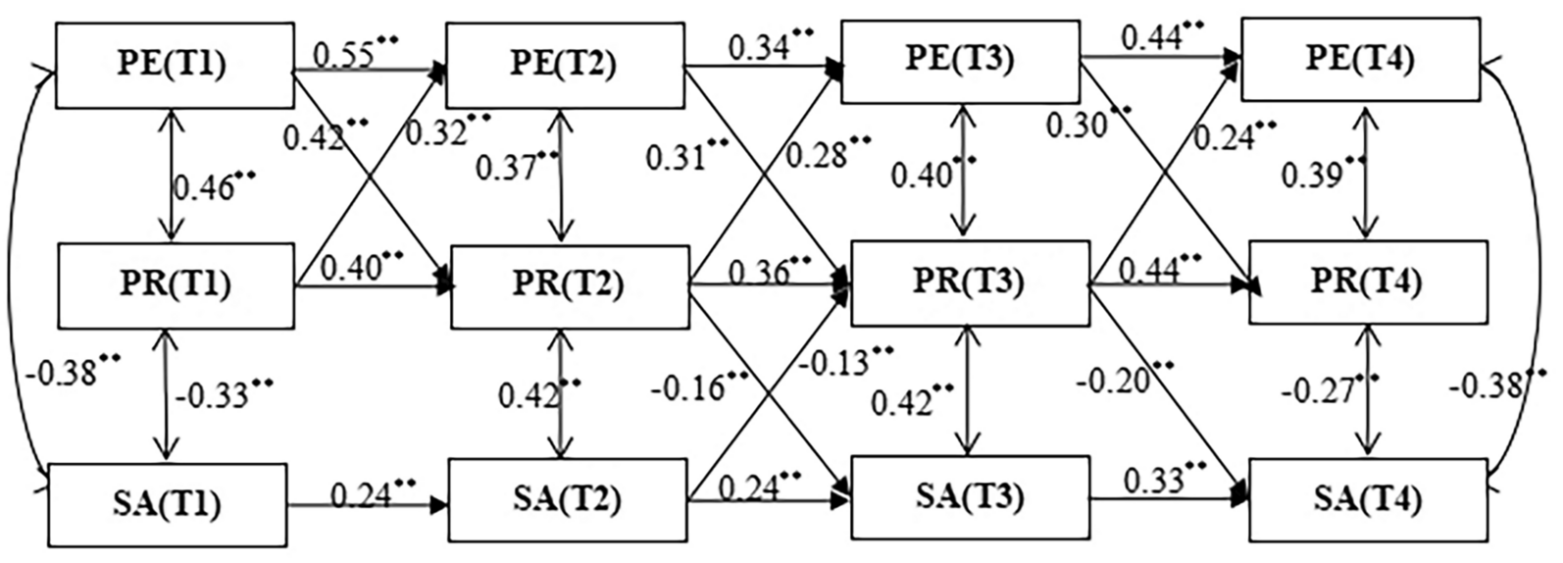
Cross-Lagged Mediating Effects among Physical Exercise, Psychological Resilience, and Social Anxiety Physical Exercise abbreviated as PE, Social Anxiety abbreviated as SA, Psychological Resilience abbreviated as PR

Figure 4 shows the longitudinal cross-lagged effects among physical exercise, psychological resilience, and social anxiety. Firstly, physical exercise has a significant positive predictive effect on psychological resilience. For instance, the path coefficient from PET1 to PR (T2) is 0.42, from PE (T2) to PR (T3) is 0.31, and from PET3 to PR (T4) is 0.30 (all *p* < 0.001), indicating that physical exercise can steadily enhance adolescents' psychological resilience at different time points. Additionally, the cross-temporal path coefficients for physical exercise are all greater than 0.34, indicating a moderate degree of longitudinal cross-temporal stability across different periods. Similarly, psychological resilience shows similar stability, with path coefficients from PR (T1) to PR (T4) all greater than 0.36, suggesting moderate longitudinal stability across different periods. For social anxiety, the path coefficients from SA(T1) to SA(T4) are all greater than 0.24, demonstrating moderate cross-temporal stability across different periods. Secondly, psychological resilience can significantly predict changes in adolescents’ social anxiety, with the path coefficient from PR (T2) to SA(T3) being − 0.16 and from PR (T3) to SAT4 being − 0.20 (both *p* < 0.001), indicating that higher psychological resilience is associated with lower levels of social anxiety in the subsequent period.

Table 3 presents the cross-lagged mediating effect values of physical exercise (PE) indirectly affecting adolescents’ social anxiety (SA) through psychological resilience (PR), further validating the mediating role of psychological resilience in this process. Initially, the mediating effect at T1 is 0.122, with a bootstrap standard error (BootSE) of 0.024 and a 95% confidence interval of [0.037, 0.281], accounting for an effect size of 37.28%; at T2, the mediating effect is 0.098, with a BootSE of 0.020 and a 95% confidence interval of [0.025, 0.215], representing an effect size of 32.10%; at T3, the mediating effect is 0.135, with a BootSE of 0.028 and a 95% confidence interval of [0.045, 0.305], corresponding to an effect size of 39.12%; at T4, the mediating effect is 0.110, with a BootSE of 0.023 and a 95% confidence interval of [0.030, 0.250], indicating an effect size of 35.14%.The total effects at each time point are 0.35, 0.30, 0.40, and 0.33, respectively, while the direct effects are 0.23, 0.20, 0.27, and 0.22. The results suggest that across different time periods, psychological resilience significantly mediates the relationship between physical exercise and social anxiety, with mediating effect values all exceeding 30%, indicating that psychological resilience is one of the important pathways through which physical exercise influences social anxiety. Overall, physical exercise not only directly reduces adolescents’ social anxiety but also alleviates social anxiety by enhancing psychological resilience, with this indirect effect showing a relatively stable role across time periods.

**Table 3:** Cross-lagged mediating effect values

Time Point	Pathway	Effect	Boot SE	BootLLCI	BootULCI	Total effect	Direct effect	Effect size %
T1	PE(T1)—> PR(T2)—> SA(T3)	0.122	0.024	0.037	0.281	0.35	0.23	37.28
T2	PE(T2)—> PR(T3)—> SA(T4)	0.098	0.020	0.025	0.215	0.30	0.20	32.10
T3	PE(T3)—> PR(T4)—> SA(T4)	0.135	0.028	0.045	0.305	0.40	0.27	39.12
T4	PE(T1)—> PR(T2)—> SA(T4)	0.110	0.023	0.030	0.250	0.33	0.22	35.14

Physical Exercise abbreviated as PE, Social Anxiety abbreviated as SA, Psychological Resilience abbreviated as PR

## Discussion

This study conducted a 1 year, four-wave longitudinal tracking of Chinese adolescents and found that physical exercise has a significant and sustained negative predictive effect on adolescent social anxiety, with psychological resilience playing a mediating role in this process. Specifically, physical exercise not only directly reduced adolescents’ social anxiety but also alleviated anxiety by enhancing psychological resilience.

### Cross-lagged effects between physical exercise and adolescent social anxiety

This study utilized cross-lagged models to investigate the longitudinal link between physical exercise and adolescent social anxiety, revealing a significant and stable negative predictive effect.

The negative predictive coefficients of physical exercise on subsequent social anxiety were − 0.31, − 0.39, and − 0.26, indicating that increased physical activity at earlier time points was associated with reduced social anxiety later on. This aligns with prior research [78, 79], which suggests that exercise improves emotional states and decreases anxiety by stimulating neurotransmitter release, such as endorphins and dopamine [80]. The study also highlights age-related nuances: younger adolescents (10–12 years) may benefit more from anxiety reduction due to self-esteem development, while older adolescents (13–15 years) might see reduced benefits due to social pressures [81]. The findings underscore the consistent and enduring impact of physical exercise on alleviating social anxiety across different time points in adolescent populations.

From a physiological standpoint, physical exercise reduces cortisol levels and mitigates stress-related physiological reactions [82], thereby significantly negatively predicting social anxiety [83]. This physiological mechanism explains the study’s findings that physical exercise has a substantial negative predictive effect on social anxiety. Exercise not only calms the nervous system, promoting composure in social contexts, but also helps adolescents develop stress-coping mechanisms through regular activity. Enhanced coping strategies reduce excessive worry about negative social evaluations, effectively lowering social anxiety [13]. Moreover, physical activity fosters social interaction, providing opportunities for peer support and recognition in team sports, which boosts self-confidence and decreases social avoidance behaviors [84].

However, the study also discovered a modest reverse predictive effect of social anxiety on physical exercise, with negative coefficients of − 0.11, − 0.02, and − 0.03 at each time point, reflecting a weak dampening influence. This indicates that social anxiety slightly impacts adolescents' engagement in physical activities, but not significantly. This aligns with research suggesting that while social anxiety might curb sports participation due to interaction fears, the positive aspects of exercise can mitigate this to an extent [33] and is supported by earlier studies [85]. Social dynamics further modify these effects: younger adolescents benefit from peer reinforcement, bolstering their self-efficacy, while older ones might face increased anxiety due to peer scrutiny, even with exercise [86]. The minimal negative impact of social anxiety on exercise in this study suggests that physical activity can, to some degree, “neutralize” the adverse effects of social anxiety.

For a deeper grasp of this dynamic, it’s crucial to recognize that social anxiety can deter physical exercise by diminishing self-efficacy. Bandura’s self-efficacy theory posits that an individual's confidence is tightly linked to their activity engagement [87]. Consequently, while social anxiety may inhibit physical activity, the self-efficacy boosted by exercise can counteract this effect. This implies that exercise not only eases social anxiety symptoms but also enhances adolescents’ confidence, prompting more vigorous engagement in physical activities, in line with Bandura’s theory [88].

The study findings also reveal that physical exercise exhibits robust cross-temporal stability, with high correlation coefficients between physical activity levels at various time points. PET1 shows significant correlations with PET2, PET3, and PET4 at 0.418, 0.359, and 0.362 respectively (p < 0.001), suggesting that adolescents who are more active at earlier stages tend to maintain high levels of physical exercise later on. This aligns with research indicating that once established, exercise habits are stable and long-lasting [89]. The findings advocate for ongoing encouragement of sports participation among adolescents at school and home, fostering good exercise habits early on to safeguard their mental health in the long term. In the context of Chinese culture, societal expectations and performance pressures can amplify adolescents’ social anxiety, potentially creating psychological barriers to physical activity due to fears of poor performance in front of peers [90]. Thus, while the study demonstrates that physical exercise significantly alleviates social anxiety, cultural factors may modulate the intensity and expression of this relationship.

Social anxiety also exhibits notable cross-temporal stability, as evidenced by the strong correlations between SAT1 and subsequent time points SAT2, SAT3, and SAT4, with coefficients of 0.269, 0.235, and 0.299 respectively (p < 0.001). This consistency over time is corroborated by previous research emphasizing the critical nature of early intervention [91]. It suggests that once social anxiety is established in adolescents, it is challenging to eradicate through brief interventions. Hence, preemptive measures are crucial. By employing strategies such as physical exercise, it is possible to mitigate anxiety symptoms before they profoundly impact adolescents' daily lives. This underscores the enduring value of physical exercise in both the prevention and intervention of social anxiety.

In conclusion, this study confirms the substantial alleviating effect of physical exercise on adolescent social anxiety and highlights the proactive role of exercise in mitigating these symptoms. While social anxiety does exert a minor inhibitory effect on physical activity, the positive impact of exercise on reducing social anxiety is predominant. Future research should delve deeper into the bidirectional relationship between social anxiety and physical exercise to enhance our understanding of this intricate interplay, as underscored by previous studies [92].

### The cross-lagged mediating role of psychological resilience

The role of sociocultural factors in shaping adolescents' psychological resilience is pivotal, with research highlighting how cultural contexts influence coping mechanisms [93]. In China’s collectivist setting, adolescents are encouraged to form support networks through group activities, which bolster psychological resilience and foster a positive environment for physical exercise, consequently reducing social anxiety. This study uses longitudinal data modeling to investigate the mediating role of psychological resilience between physical exercise and social anxiety, confirming that exercise can both directly decrease social anxiety and indirectly affect it by enhancing resilience. Younger adolescents often rely on external support, such as from family and teachers, while older ones develop intrinsic resilience through self-reflection. This age-related difference underscores the need for targeted interventions to foster psychological resilience. For instance, Alessandri et al. [57] note that resilience develops from late adolescence into early adulthood, suggesting that interventions should be tailored: team sports for middle schoolers to build social support, and autonomous exercise opportunities for high schoolers to increase self-efficacy [94]. This study’s longitudinal approach, compared to cross-sectional studies, offers a dynamic view on the long-term causality between exercise, resilience, and social anxiety. Analysis shows that exercise significantly predicts psychological resilience and social anxiety at different time points, with increased physical activity associated with higher resilience and lower social anxiety in subsequent periods. This aligns with research on the positive effects of physical exercise on adolescent mental health [95], reinforcing the efficacy of exercise in mitigating adolescent anxiety.

The data show that the positive predictive effect of physical exercise on psychological resilience is significant at different time points, such as the path coefficient from PET1 to PRT2 being 0.42, and from PET2 to PRT3 being 0.31 (p < 0.01), indicating that physical exercise significantly promotes psychological resilience at all times. This result aligns with previous scholarly findings that physical exercise can enhance adolescents' self-efficacy, thereby increasing psychological resilience [96].Regarding the relationship between psychological resilience and social anxiety, the negative predictive effect of psychological resilience is also confirmed. The data indicate that the path coefficient from PRT2 to SAT3 is − 0.16, and from PRT3 to SAT4 is − 0.20 (both p < 0.001), suggesting that higher psychological resilience is associated with lower levels of social anxiety in adolescents at the next time point. This is consistent with earlier research [97], indicating that psychological resilience, as an important psychological resource, can effectively buffer the negative impact of external stress on individual mental health and plays a significant role in coping with social anxiety.

Additionally, it is noteworthy that the study robustly establishes the direct impact of physical exercise on reducing adolescent social anxiety, with significant negative predictive effects observed across all time points. For instance, the path coefficients from PET1 to SAT2 (− 0.31) and PET2 to SAT3 (− 0.39) underscore the efficacy of exercise in diminishing anxiety levels, aligning with Chinese cross-sectional studies that link physical activity with lower social anxiety in adolescents [98]. The longitudinal data reinforce the conclusion that exercise has both immediate and long-term benefits in curbing social anxiety through the establishment of exercise habits.

Conversely, the reverse effect of social anxiety on physical exercise is found to be weak, with path coefficients from SAT1 to PET2 (− 0.11), SAT2 to PET3 (− 0.02), and SAT3 to PET4 (− 0.03) indicating a minimal inhibitory impact. This suggests that while social anxiety might slightly deter exercise participation, the anxiety-alleviating effects of physical exercise remain pronounced, reinforcing the intervention evidence for adolescent social anxiety.

The study also highlights the significant mediating role of psychological resilience between physical exercise and social anxiety, with Bootstrap analysis showing mediation effect values of 0.122 (T1), 0.098 (T2), and 0.135 (T3), all with 95% confidence intervals excluding zero. This aligns with compensation theories in organizational management [99], indicating that exercise reduces anxiety both directly and indirectly by fulfilling psychological needs and enhancing resilience. This perspective enriches our understanding of how exercise intervenes in adolescent social anxiety by emphasizing the mediating role of psychological resilience.

In conclusion, this longitudinal study affirms the substantial role of physical exercise in mitigating adolescent social anxiety, particularly through the enhancement of psychological resilience. It provides a dynamic view of the interplay between physical exercise, resilience, and social anxiety, offering a more holistic theoretical foundation for interventions in adolescent social anxiety compared to previous cross-sectional research.

### Research contributions, limitations, and future directions

This study systematically investigates how physical exercise alleviates adolescent social anxiety through four-time point longitudinal tracking, focusing on the mediating role of psychological resilience. Unlike previous cross-sectional studies, it demonstrates that physical exercise not only directly reduces social anxiety at each time point but also indirectly lowers anxiety levels in social situations by enhancing adolescents' psychological resilience. This finding provides empirical support for the long-term effectiveness of physical exercise as a psychological intervention, confirming its role in strengthening individuals’ resilience and coping abilities against social stress. The study enriches the theoretical framework linking physical exercise and mental health and offers practical insights for educational, familial, and social interventions, highlighting the importance of physical activity in promoting adolescent mental health.

Despite the strengths of the longitudinal design, there are limitations to consider. First, while the four time points reveal short-term trends in physical exercise, psychological resilience, and social anxiety, the study's duration is relatively limited and does not fully capture the long-term cumulative effects of physical exercise. Future research should extend the tracking period to explore these long-term impacts. Second, the sample is drawn from specific cities in China; although large and diverse in age, the generalizability of the findings to other cultural contexts requires further validation. Lastly, reliance on self-reported data may introduce social desirability bias or memory inaccuracies; future studies should incorporate objective measures or physiological indicators (such as heart rate and cortisol levels) to enhance data accuracy.

Future research can expand in several directions. First, extending the study's timeframe to investigate the long-term effects of physical exercise on psychological resilience and social anxiety, particularly whether sustained activity leads to more stable resilience and lasting anxiety relief. Second, distinguishing between types and intensities of physical exercise to assess their differential impacts on resilience and social anxiety. Lastly, verifying these findings in cross-cultural contexts to explore the universality and specificity of physical exercise's effects on adolescent mental health, thereby providing broader theoretical and practical support for global adolescent mental health promotion.

## Conclusion

The main conclusions of the study are as follows: (1) Physical exercise and psychological resilience show a stable upward trend, while social anxiety shows a stable downward trend; (2) Physical exercise can significantly negatively predict social anxiety in the next period, and the two variables have a stable cross-time effect; (3) Psychological resilience plays a mediating effect in the impact of adolescent physical exercise on social anxiety, and the stability across time is reliable. Specifically, in measurements across multiple time points, physical exercise can significantly negatively predict social anxiety, with psychological resilience playing a significant mediating role, effectively curbing the occurrence of adolescent social anxiety.

## Data Availability

The data used in this study have been anonymized and securely stored. The research team will provide data for academic research upon reasonable request and can be applied for via email to the corresponding author, Wu Jingtao.
